# Navigating Through Technical Difficulties in Operating Massive Cervical Fibroid—A Case Report and Narrative Review

**DOI:** 10.1155/crog/8529785

**Published:** 2026-01-28

**Authors:** Alfonsus Zeus Suryawan, Evita Laras Dewayanti, Dini Pusianawati

**Affiliations:** ^1^ Department of Obstetrics and Gynecology, Padjadjaran University, Hasan Sadikin General Hospital, Bandung, West Java, Indonesia, unpad.ac.id

**Keywords:** cervical fibroid, cervical ring, extrafascial hysterectomy, intrafascial hysterectomy, ureter injury

## Abstract

**Introduction:**

Total abdominal hysterectomy (TAH), commonly referred to as extrafascial hysterectomy, is widely used in standard gynecological practice. However, hysterectomy performed in cervical leiomyoma presents greater challenges and pitfalls that require a modified approach. This case report wishes to highlight the importance of anatomical identification and the application of the intrafascial approach of hysterectomy.

**Case Report:**

A 57‐year‐old multiparous woman was admitted with a significant abdominal mass. During the physical examination, a large globular firm mass, corresponding to approximately 24 weeks of pregnancy, was identified. Ultrasonography revealed a cervical fibroid, and subsequent biopsy confirmed this diagnosis. We decided to perform intrafascial hysterectomy. Intraoperatively, the cervical fibroid was found 20 × 16 cm in size, whereas the uterus measured 14 × 14 cm. Pathological examination confirmed the presence of leiomyoma and endocervicosis of the cervix.

**Discussion:**

Cervical fibroids present considerable challenges during surgical procedures due to alterations in uterine anatomy, which can affect the ureter′s danger zone. The application of the intrafascial technique in hysterectomy is essential for minimizing the risk to the ureters and for preserving the cervical ring.

## 1. Introduction

Hysterectomy has historically been the tried‐and‐true procedure for the treatment of uterine fibroids and remains the only proven permanent solution, particularly when other treatments have proven ineffective or for those not desiring future fertility [1, 2]. The current common approach to hysterectomy is extrafascial hysterectomy, in which the cervical ring and endopelvic fascia are removed along with the uterus. This technique has demonstrated effectiveness in removing benign and malignant masses while preserving minimal uterine tissue [3–5]. However, in cases of cervical fibroids (LO8), especially the large one, it can be quite challenging to identify the uterosacral–cardinal complex and vaginal borders due to significant anatomical distortion. Additionally, these types of fibroids present difficulties in identifying the ureter, which runs alongside the uterine artery near the uterosacral–cardinal complex (12 mm) [6, 7].

## 2. Case Report

A 57‐year‐old female came to gynecology polyclinic with a referral from district hospital diagnosed as a suspected ovarian malignancy. The patient reported a progressive enlargement of an abdominal mass over the past year, initially palpable near the symphysis pubis and currently palpable at the level of the umbilicus. The patient also stated an increase in abdominal pain, which was characterized as sudden yet brief and occurring intermittently. Difficulty in urination was present in the last 2 months before admission. The patient had menopause at 47 years old; however, she complained of scant bleeding from her birth canal this month with no history of intercourse nor trauma.

At physical examination, a large globular, firm mass with well‐defined margins, nontender with restricted mobility, corresponding to 24 weeks gravid uterus was found. Speculum examination demonstrated that the cervix was notably displaced toward the anterior fornix due to the mass occupying approximately one‐third of the upper vagina. Ultrasonography revealed a mass in the cervical region measuring 9.98 × 20.74 cm, associated with an enlarged uterus (Figure 1). A cervical biopsy confirmed the presence of a cervical fibroid.

Figure 1Ultrasonography of the mass; (a) mass position below uterus and (b) cervical mass measuring 9.98 × 20.74 cm.

**Figure:**
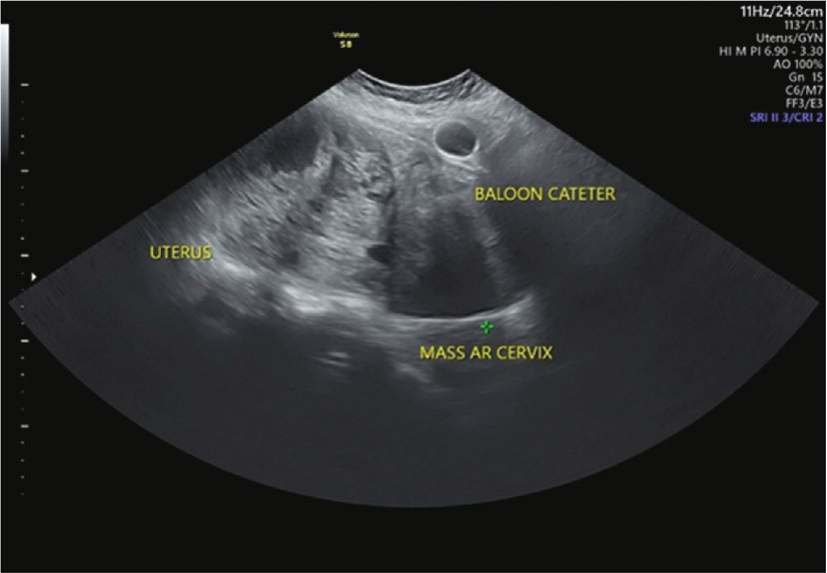
(a)

**Figure:**
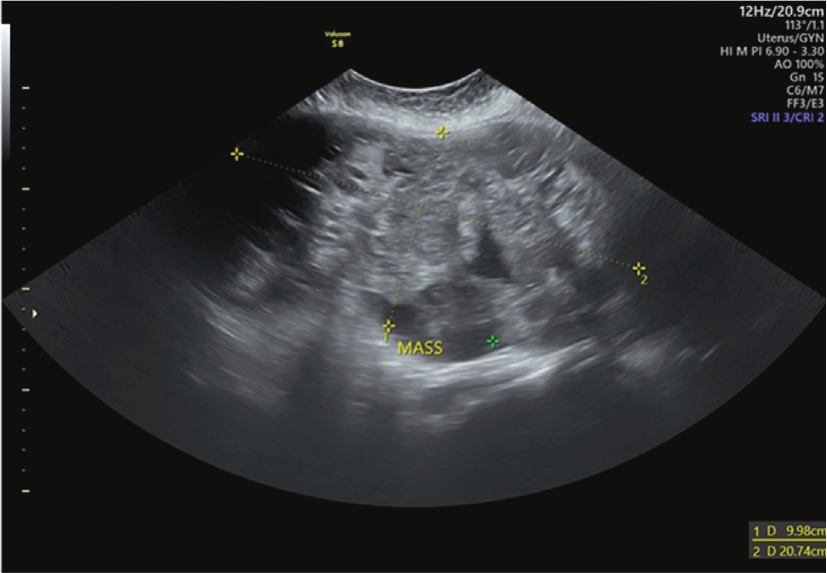
(b)

**Figure 2:**
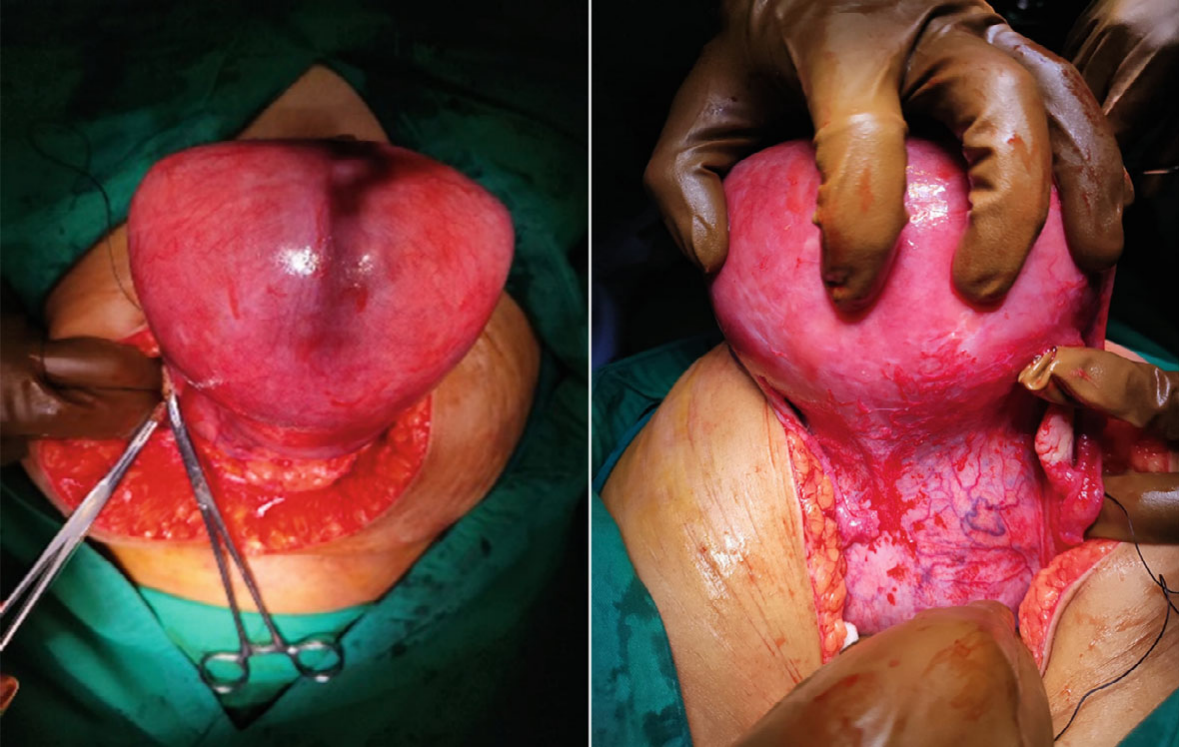
Anterior and posterior view of uterus during operation.

The patient was scheduled for a hysterectomy and bilateral salpingo‐oophorectomy. During the procedure, after the peritoneum was opened, it was noted that the uterus was enlarged to approximately the size of a 20‐week gestation and fibroid masses were identified in both the fundus and cervical region (see Figure 2). The hysterectomy was performed step‐by‐step until the ligation of the uterine artery. Following a reassessment of the cervical mass, which was approximately the size of the uterine fundus, it was decided that an intrafascial approach was deemed necessary to mitigate the risk of injury to adjacent structures, such as the ureter and bladder.

**Figure 3:**
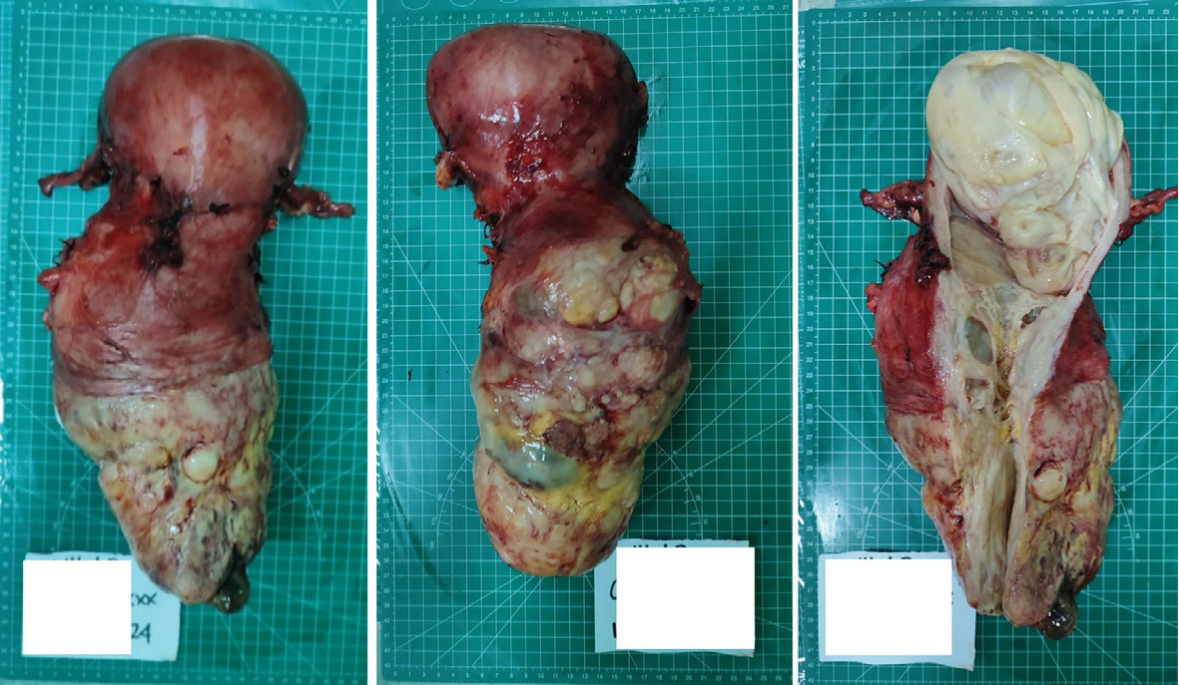
Anterior, posterior, and inside view of specimen after a successful operation.

Following the successful removal of the uterus, the specimen was measured using a specimen‐measuring board. The cervical area was found to be 20 × 16 cm, whereas the uterine fundus measured 14 × 14 cm (Figure 3). Pathological examination revealed the presence of leiomyoma and endocervicosis of the cervix. Currently, she has completed her wound care a month after the operation and controlled herself to gaynecology polyclinic.

## 3. Discussion

Leiomyomas are the most common benign gynecological tumor. Cervical fibroids constitute only 1%‐2% of all fibroids, and mostly they are situated in the supravaginal portion of the cervix [1, 8, 9]. Huge cervical fibroids, like in our case, are even rarer. A cervical leiomyoma is commonly single and is either interstitial or subserous. Rarely does it become submucous and polypoidal [2, 10]. The symptoms of cervical fibroids depend upon the type of cervical fibroid. Anterior fibroids bulge forward and undermine the bladder, whereas posterior ones flatten the pouch of Douglas backwards, compressing the rectum against the sacrum. Lateral cervical fibroids, starting on the side of the cervix, burrow out into the broad ligament and expand it. Their relation to the ureter is important. Wherever the ureter and uterine artery may be in relation to the fibroid, they will always be extracapsular. The knowledge of this fact can turn a potentially dangerous procedure into a relatively safe operation [3, 10, 11]. Central cervical fibroid expands the cervix equally in all directions, elevated on the top of which the uterus resembles *“*the lantern on the top of St. Paul′s*”* [5, 12, 13]. Base on these findings, we did not perform insertion of ureter catheter on this case. This decision is also on point with the fact that there is no evidence of urinary tract obstruction, even though the myoma is huge.

The size of cervical fibroid can also vary from a few millimeters to several centimeters. Giant cervical fibroids are considered when it exceeds a size of 9 cm or a weight of 800 g as described by Pedraza et al. [14] Although they are mainly treated surgically, currently, there is not a standardized treatment for cervical leiomyomas [6, 15]. In the last decades, many new techniques, alternative to hysterectomy with conservation of the uterus have been developed in case fertility sparing was required in the patient [16–18]. The problems anticipated during hysterectomy for large cervical fibroids are due to distortion of normal anatomy of the ureter and uterine vessels and sometimes due to pulled up bladder anteriorly. Therefore, there are more chances of injury to the ureter, bladder, and uterine vessels [19, 20]. Commonly, the technique we use in total hysterectomy is derived from Richardson technique from 1927 [1]. The technique ensures complete removal of the uterus and cervix; however, this also with reduction of pelvic support as the endopelvic fascia and uterosacral–cardinal complex were removed.

**Figure 4:**
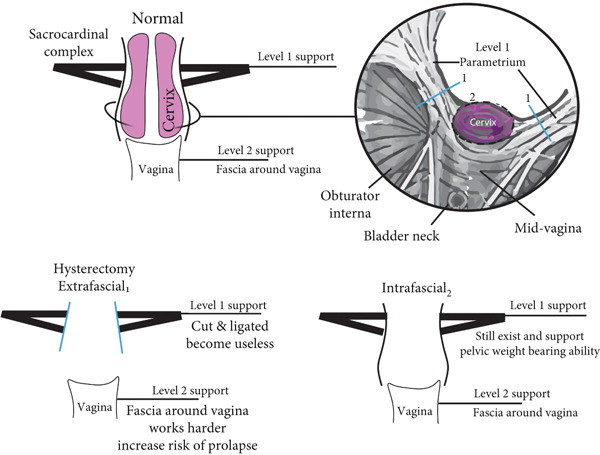
Illustration of extrafascial and intrafascial hysterectomy. (1) Marking extrafascial cutting, removing Level 1 support entirely; (2). marking intrafascial cutting, preserving pelvic ligaments structure.

This was the problem that was solved by intrafascial hysterectomy that Aldrige proposed in 1950′s [2, 3]. Looking at the pelvic support level proposition by De′Barkley, the endopelvic fascia and uterosacral–cardinal complex around the cervix classifies as Level 1 [4]. The perseverance of the cervical ring leads to lower rates of pelvic organ prolapse [5]. The preservation of the cervical ring has been associated with lower rates of pelvic organ prolapse. (Figure 4) [5].

Intrafascial hysterectomy is becoming less commonly used due to its lack of radical approach; for Stage 1A cervical cancer, we typically perform extrafascial hysterectomy as intrafascial is a less invasive technique that removes the cell mass and may address early parametrial metastases. Extrafascial also proved to be much simpler with ligation of sacrouterine complex outside cervical ring after uterine artery was identified, cut, and clamped (Figure 4). In big cervical fibroids which exceed 5 cm in diameter, extrafascial hysterectomy proves challenging anatomical distortion. Ureter, which crosses uterine artery near uterosacral–cardinal complex, is only 12 mm from uterine corpus, and enlargement of the cervical area could push the uterine body closer to the ureter [6].

Enlargement of cervical area made identification of vaginal area harder and increased risk of ureteric injury near Wertheim canal that runs alongside vesicouterine pouch on each perspective leafs. Retrograde hysterectomy also proved hard as uterosacral ligament stretches far, and it is hard to assess cervical border [7]. On this case, after uterine artery was skeletonized and ligated, we perform intrafascial approach of the uterus. As important point in intrafascial hysterectomy is not “cut down” the uterine cervix but “pull out” the uterine cervix under enough traction of the uterus [2]. After slowly reaching the bottom of the uterus and cervix, circular incision was made around the cervical fibroid neck. After the outer layer was peeled off, it is easy to pull out the uterine cervix under enough traction. This was made possible with optimal bladder positioning with identification and dissection of uterovesical plica [1].

Securing vaginal stump in big cervical fibroids is also difficult, depending on how much vagina was removed alongside the cervical mass. In this case, after cutting the estimated line of the cervical uterine outer layer at the middle area of the mass and complete mass removal, the vaginal stump was considerably big due to the distention of the fibroid. Resection of the vaginal cuff is unnecessary due to the nature of the intrafascial approach [2].

## 4. Conclusion

Cervical fibroid, even though classified as benign, posed surgical risk that is significantly higher than that of other benign conditions of the female reproductive tract. Applied surgical anatomy understanding and usage of intrafascial hysterectomy proved pivotal for successful surgery, as applied in this case. Furthermore, the intrafascial approach is safely performed even in patients with firm adhesion in Douglas′s pouch or around the uterine cervix.

## Ethics Statement

This study is exempt from ethical approval as determined by the institutional and departmental review board.

## Consent

Written informed consent was obtained from the patient for the publication of this case report and any accompanying images. A copy of the written consent is available for review by the Editor‐in‐Chief of this journal.

## Disclosure

All authors gave final approval of the version to be published; have agreed on the journal to which the article has been submitted; and agree to be accountable for all aspects of the work.

## Conflicts of Interest

The authors declare no conflicts of interest.

## Author Contributions

All authors made a significant contribution to the work reported, whether that is in the conception, study design, execution, acquisition of data, analysis and interpretation, or in all these areas; took part in drafting, revising or critically reviewing the article.

## Funding

No funding was received for this manuscript.

## Data Availability

The data that support the findings of this study are available from the corresponding author upon reasonable request.
